# Radium in Prostatic Cancer

**Published:** 1914-02

**Authors:** 


RADIUM IN PROSTATIC CANCER. O. Pasteau and Degrais of Paris, *Can. Pract & Rev.,* December, 1913, report several cases, the radium being introduced through the elbow-catheter with lumina. While several incompletely treated, recent and unsuccessful cases are mentioned, the general conclusion is that the action is favorable, inoperable growths being reduced to operable condition.

